# The Role of Manganese Superoxide Dismutase in Inflammation Defense

**DOI:** 10.4061/2011/387176

**Published:** 2011-10-03

**Authors:** Chang Li, Hai-Meng Zhou

**Affiliations:** ^1^School of Life Sciences, Tsinghua University, Beijing 100084, China; ^2^Zhejiang Provincial Key Laboratory of Applied Enzymology, Institute of Tsinghua University, Yangtze Delta Region, Jiaxing 314006, China

## Abstract

Antioxidant enzymes maintain cellular redox homeostasis. Manganese superoxide dismutase (MnSOD), an enzyme located in mitochondria, is the key enzyme that protects the energy-generating mitochondria from oxidative damage. Levels of MnSOD are reduced in many diseases, including cancer, neurodegenerative diseases, and psoriasis. Overexpression of MnSOD in tumor cells can significantly attenuate the malignant phenotype. Past studies have reported that this enzyme has the potential to be used as an anti-inflammatory agent because of its superoxide anion scavenging ability. Superoxide anions have a proinflammatory role in many diseases. Treatment of a rat model of lung pleurisy with the MnSOD mimetic MnTBAP suppressed the inflammatory response in a dose-dependent manner. In this paper, the mechanisms underlying the suppressive effects of MnSOD in inflammatory diseases are studied, and the potential applications of this enzyme and its mimetics as anti-inflammatory agents are discussed.

## 1. Introduction

Aerobic organisms utilize molecular oxygen (dioxygen; O_2_) as the final electron receptor in the oxidative phosphorylation electron transport chain. Normally, O_2_ is reduced to H_2_O after receiving four electrons; however, partial reduction of O_2_ leads to the formation of highly reactive oxygen species (ROS), including the superoxide anion (O_2_^−·^), hydrogen peroxide (H_2_O_2_), and the hydroxyl radical (OH^*·*^). ROS can damage lipids, proteins, and DNA, leading to aberrant downstream signaling or stimulation of apoptosis [1, 2]. Oxidative stress has been implicated in neurodegenerative diseases, aging, cancer, pulmonary fibrosis, and vascular diseases [3–6]; elimination of unwanted ROS is, therefore, very important for organismal survival. To confront oxidative stress caused by ROS, organisms have evolved a variety of antioxidant enzymes, such as superoxide dismutase, catalase, and glutathione peroxidase. However, ROS can also act as cell signaling molecules and cause damage to foreign bodies [2]. ROS are, therefore, double-edged swords with respect to biological processes. 

Inflammation is a host defense response to infectious agents, injury, and tissue ischemia. Inflammation occurs because of lymphocyte and macrophage invasion and the secretion of mediators of inflammation such as cytokines, cyclooxygenase products, and kinins [7]. Inappropriate inflammation is a hallmark of various diseases. A large body of evidence suggests that antioxidant enzymes are key regulators of inflammation. Manganese superoxide dismutase (MnSOD) is an enzyme present in mitochondria that is one of the first in a chain of enzymes to mediate the ROS generated by the partial reduction of O_2_. MnSOD has been implicated in a number of oxidative stress-related diseases. In this paper, we will discuss the role of MnSOD in various inflammation-associated diseases and explore the therapeutic potentials of agents that regulate its expression.

## 2. Regulation of MnSOD

MnSOD mRNA levels can be upregulated by several factors: LPS [8], cytokines such as TNF [9], IL-1 [10], and VEGF [11], UVB irradiation, ROS [12], and thioredoxin [13]. The human MnSOD gene (*sod2*) has a housekeeping promoter with multiple copies of Sp-1- and AP-2- binding sequences. The promoter region also contains a GC-rich region and NF-*κ*B transcription regulation elements [14]. Several enhancers are also present in the promoter region and in the second intron [15]. TNF and IL-1 inductions of *sod2 *mRNA require a 238-bp TNF response element (TNFRE), which is located in intron 2. Both C/EBP and NF-*κ*B bind to the TNFRE enhancer to interact with the *sod2* promoter, resulting in the upregulation of MnSOD transcription [9]. TPA-induced MnSOD expression is due to the transcription factor specificity protein 1- (SP1-) mediated PKC signaling [16]. Dimeric SP1 can bind to GC-rich sequences of GGGCGG, but the binding affinity and transcription properties vary according to the interacting cofactors [17–19]. 

The downregulation of mRNA levels is as important in biological processes as is upregulation. Because ROS can act as intracellular secondary messengers, maintaining proper levels of these molecules is important for normal cellular function. This suggests that antioxidant enzymes are likely maintained at low levels in cells. 

Many studies have reported the downregulation of MnSOD mRNA levels in disease states. Many tumor cell lines have mutations in the promoter region of the MnSOD gene that increase the number of AP-2-binding sites. AP-2 can interact with SP-1 within the promoter region and decrease promoter activity, thus downregulating transcription [17]. VEGF can upregulate MnSOD mRNA levels through the ROS-sensitive PKC-NF-*κ*B and PI3K-Akt-Forkhead signaling pathways [11]. FOXO3a is a member of the Forkhead family of transcription factors. Phosphorylation of Ser253 of FOXO3a decreases DNA binding and consequently gene expression, which results in the age-related activation of Akt [18]. 

Aging-related disorders are often associated with oxidative stress. Epigenetic silencing of the MnSOD gene has also been observed in human breast cancers. Both DNA methylation and histone modification contribute to this regulation [19]. Epigenetic modification influences the abilities of SP1, AP-1, and NF-*κ*B to bind to cis-elements in the promoter region of the MnSOD gene, resulting in silencing of this gene. 

MnSOD mRNA upregulation always results in increased levels of MnSOD protein [20]. MnSOD is located in mitochondria; therefore, its major role appears to be controlling the levels of O_2_^−·^ in mitochondria. H_2_O_2_ is a product of MnSOD-catalyzed reactions; increased MnSOD activity results in H_2_O_2 _ accumulation. H_2_O_2 _ can act as a second messenger or as a Fenton reaction agent, thereby causing damage to cells. To elucidate the significance of MnSOD regulation, the function of MnSOD must be considered.

## 3. The Function of MnSOD

In cancer cells, MnSOD is almost always suppressed by certain transcription factors or through epigenetic modification of cis-elements or chromatin. Overexpression of MnSOD in cancer cells can alter the phenotype in culture; the cells lose the ability to form colonies, a trait characteristic of malignant cells [21]. A large number of studies have reported that ROS play an important role in tumor metastasis [22, 23]. ROS can activate cell signaling pathways and/or mutate DNA, thereby promoting tumor proliferation and metastasis. This may explain why tumor cells almost always express MnSOD at low levels. Exogenous MnSOD can block ROS signaling to inhibit tumorigenesis, suggesting that MnSOD may be a potential antitumor therapeutic target. Overexpression of MnSOD can enhance the activity of the superoxide-sensitive enzyme aconitase and inhibit pyruvate carboxylase activity, thereby altering the metabolic ability of the cell and inhibiting cell growth [24].

A mouse knockout model of manganese superoxide dismutase has proven to be a useful model for elucidating the function of MnSOD. As stated previously, the major function of MnSOD is to protect mitochondria from ROS damage. However, although ROS can damage organisms, they are also mediators of cell signaling. Developing mice fetuses lacking manganese superoxide dismutase do not survive to birth; overexpressions of other types of SOD cannot attenuate this symptom [25]. Newlyborn MnSOD knockout mice have extensive mitochondrial injuries in multiple tissues. Disorders such as Leigh's disease and Canavan disease are characterized by mitochondrial abnormalities. Reductions in the levels of a variety of energy metabolism enzymes, especially those with a role in the TCA cycle, have also been noted in these disorders. Treatment of *Sod2* ^tm1cje^(−/−) mutant mice with the manganese superoxide dismutase mimetic manganese 5,10,15,20-tetrakis (4-benzoic acid) porphyrin (MnTBAP) improved these mice and dramatically prolonged their survival times [26–28]. 

Miki et al. studied the cytological differences between wild-type mice and heterozygous *sod2* knockout (*sod2 *−/+) mice after permanent focal cerebral ischemia (FCI). Cytochrome c accumulated at an early stage and was significantly more elevated in *sod2* −/+ mice than it was in wild-type mice. A remarkable increase in DNA laddering was also observed in the *sod2* −/+ mice but not in the wild-type mice, suggesting that MnSOD can block the release of cytosolic cytochrome c and prevent apoptosis [29]. Neurotoxins such as 1-methyl-4-phenyl-1,2,5,6-tetrahydropyridine (MPTP), 3-nitropropionic acid (3-NP), and malonate are commonly used in neurodegenerative functional models. Mice with a partial deficiency in MnSOD are more sensitive to these mitochondrial toxins than are normal mice [30], suggesting that MnSOD is an antitoxin agent that scavenges free radicals generated by environmental toxins that may cause neurodegeneration.

MCF-7 human carcinoma cells exposed to single-dose radiation and radioresistant variants isolated from MCF-7 cells following fractionated ionizing radiation (MCF and FIR cells) were found to possess elevated MnSOD mRNA levels, activity, and immunoreactive proteins. MnSOD-silenced cells were sensitive to radiation. The genes P21, Myc, 14-3-3 zeta, cyclin A, cyclin B1, and GADD153 were overexpressed in both MCF + FIR and MCF + SOD cells (MCF-7 cells overexpressing MnSOD). These genes were suppressed in *Sod2* knockout mice (−/−) and in MnSOD-silenced cells [31]. These six genes are survival genes [32–34] that protect cell from radiation-induced apoptosis.

## 4. MnSOD in Diseases with Inflammation

**Figure 1:**
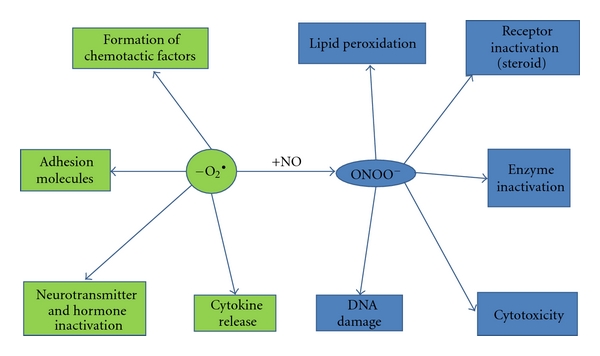
Biological basis and effects of superoxide generation. Excessive production of superoxide anions can lead to inflammation through many pathways, such as generation of peroxynitrite.

Inflammation is a complex response to harmful stimuli, such as tissue injury, pathogens, autoimmune damage, ischemia and other irritants [35]. Numerous inflammation-associated molecules and cells remove injurious stimuli and repair damaged tissues. The healing process includes the destruction of “foreign objects” and the repair of injured self-tissues. If targeted destruction and associated repair are not correctly programmed, inflammatory disorders resulting in diseases such as psoriasis, inflammatory bowel disease, and neurodegenerative diseases develop [36, 37]. Superoxide anions have proinflammatory roles, causing lipid peroxidation and oxidation, DNA damage, peroxynitrite ion formation, and recruitment of neutrophils to sites of inflammation [38–40]. Elimination of superoxide anions by MnSOD and its isoenzymes can, therefore, be considered to be anti-inflammatory (Figure 1).

Inflammatory bowel disease (IBD) is accompanied by the excessive productions of reactive oxygen and nitrogen metabolites [41]. The concentration of malondialdehyde (MDA), which can serve as an index for lipid peroxidation, was found to be increased in inflamed mucosa cells [42]. Lipid peroxidation is associated with hydroxyl radicals and superoxide anions. In inflamed cells, levels of MnSOD are suppressed relative to those of normal cells, indicating that MnSOD may be a therapeutic target. NOD2 is a susceptibility gene for IBD; the NOD2 protein can activate the immune system by triggering NF-*κ*B and can negatively regulate the Toll-like receptor-mediated T-helper type 1 response, thereby increasing susceptibility to infection [43, 44]. The pathology of IBD requires further investigation. Currently, drugs targeting NF-*κ*B or ROS have been found to be somewhat effective.

The skin is the largest organ of the human body and acts as a physical boundary to protect the internal organs against the environment. Skin dysfunction could result in injury to deeper tissues. Skin injuries can activate the acute inflammatory response, and infection can heighten this response. Psoriasis is a chronic disease characterized by inflamed, scaly, and frequently disfiguring skin lesions. Epidermal keratinocytes in this disease show altered differentiation and hyperproliferation, and immune cells such as T-cells and neutrophils are present at lesion sites [45]. JunB is a component of the AP-1 transcription factor complex that regulates cell proliferation, differentiation, the stress response, and cytokine expression [46]. Both JunB and c-Jun are highly expressed in lesional skin, but levels of JunB have been shown to be low in severe psoriasis and intermediate in mild psoriasis, while c-Jun is expressed in the opposite manner [47]. 

Most components of the AP-1 transcription factor are redox-sensitive proteins regulated by ROS signaling. Exposure of keratinocytes to chemical irritants, allergens, or inflammatory stimuli triggers activation of several stress-sensitive protein kinases that are mediated by ROS. ROS enhance EGFR phosphorylation and activate ERKs and JNKs [48]. ROS also activate NF-*κ*B during skin inflammation. These findings indicate that antioxidant enzymes may have potential as therapeutic agents. 

MnSOD was found to be highly expressed in psoriasis, but this expression was not associated with the pathology of psoriasis [49]. A reasonable hypothesis is that lesional skin cells are induced to express MnSOD by cytokines released from inflammatory cells in order to counteract inflammation-induced oxidative stress. Although native MnSOD has shown promising anti-inflammatory properties against many diseases in both preclinical and clinical studies, there are several drawbacks to using native MnSOD as a therapeutic agent and pharmacological tool. Low molecular weight mimetics of SOD were, therefore, developed to address some of the drawbacks of native SOD use. 

**Figure 2:**
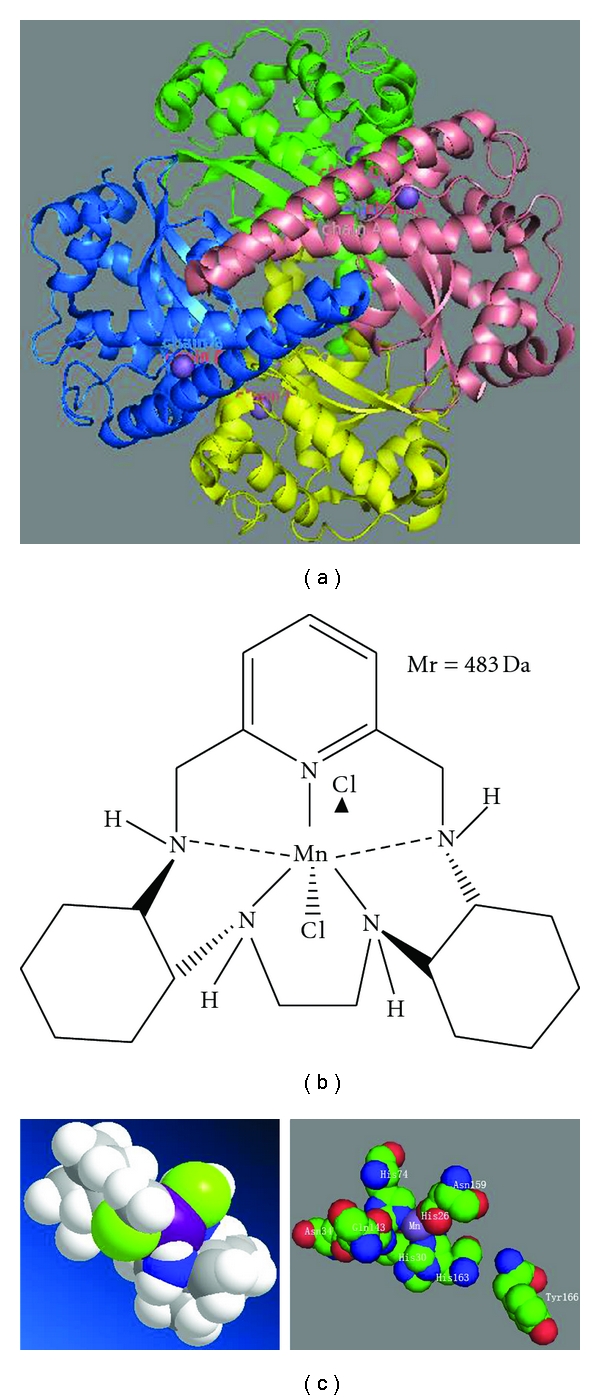
The three-dimensional (3D) structure of human manganese superoxide dismutase (a) and that of the synthetic superoxide dismutase mimetic M40403 (b). The 3D structure of M40403 and the active site of MnSOD (c). This manganese-containing biscyclohexylpyridine has superior catalytic activity compared to that of the native enzyme. Note that Mn^2+^ is purple and Cl^−^ is light green in the 3D structure of M40403.

To date, frequently used SOD mimetics are MnTBAP, the Mn(III)-salen complex, and Mn II-pentaazamacrocyclic ligand-based SOD mimetics [50]. In a mouse model of lung pleurisy, treatment with MnTBAP before carrageenan administration was found to suppress inflammatory responses in a dose-dependent manner [51]. The mechanism of attenuation of inflammation by SOD mimetics is the reduction of peroxynitrite formation through the elimination of superoxide anions before they react with nitric oxide. Because peroxynitrites are numerous and have pro-inflammatory and cytotoxic effects, administration of SOD mimetics is clinically very important. M40403 (Figure 2) was derived from 1,4,7,10,13-pentaazacyclopentadecane containing added bis(cyclohexylpyridine) functionalities. It is the best products achieved high stability and catalytical activity based on the computer-aided design. M40403 gets a high specificity for scavenging superoxide anion, while other oxidants, such as hydrogen peroxide, peroxynitrite, and hypochlorite, are hardly oxidative to Mn-II packaged in the complex. The biological function of M40403 has been tested in several models [52]. The global mechanism seems that M40403 could block nitrosation of tyrosine in proteins, indicating that superoxide anion driven formation of peroxynitrite might be responsible for the nitrosation. While increasing evidences are suggesting that nitrosation plays important role in many inflammation-related diseases [50, 53], this low molecular mass synthetic is a potential therapeutic agent for curing inflammation.

## 5. Conclusions

Inflammation is a traditional but complex problem that still requires extensive investigation. ROS play a very important role in the triggering and promotion of inflammation. Thus, antioxidant enzymes that can function as ROS scavengers are ideal therapeutic agents. Data generated from mouse models have shown that native MnSOD has anti-inflammatory properties but also some practical disadvantages. MnSOD mimetics were, therefore, developed to address the shortcomings of native MnSOD. These low molecular weight molecules have been tested in several *in vivo* and *in vitro *models; they have all been shown to be effective mimics of SOD. Despite the great achievements made over the past few decades, however, there is still a need to develop even more efficient and compatible anti-inflammatory agents suitable for clinical pharmaceutical therapy.

## Abbreviations

MnSOD: Manganese superoxide dismutase;
ROS: Reactive oxygen species;
MnTBAP: Manganese 5,10,15,20-tetrakis (4-benzoic acid) porphyrin.
